# MSTFFNet: Multi-Scale Time-Frequency Fusion with Self-Estimated SNR Conditioning for Robust Automatic Modulation Recognition

**DOI:** 10.3390/s26165208

**Published:** 2026-08-17

**Authors:** Zhiyuan Wu, Xin Xiang, Pengyu Dong, Rui Wang, Guo Xiao

**Affiliations:** 1 Graduate School, Air Force Engineering University, Xi’an 710038, China; wws_hf0612@163.com (Z.W.); xiaog1097@163.com (G.X.); 2 Aviation Engineering School, Air Force Engineering University, Xi’an 710038, China; xxisdn2002@sina.com (X.X.); 15353724115@163.com (R.W.)

**Keywords:** automatic modulation recognition, deep learning, time-frequency fusion, SNR self-estimation, signal classification

## Abstract

Automatic modulation recognition (AMR) identifies the modulation scheme of received radio frequency (RF) signals under unknown channel conditions and underpins spectrum monitoring and signal demodulation in wireless systems. Under low signal-to-noise ratio (SNR), multipath fading, and limited observation length, however, the discriminative features of modulated signals are severely attenuated, degrading recognition robustness. We propose MSTFFNet, a multi-scale time-frequency fusion network that addresses these challenges with two designs. First, it fuses the raw in-phase/quadrature (I/Q) signal with its short-time Fourier transform (STFT) time-frequency map at the token level through dual-stream heterogeneous encoding, capturing complementary temporal and spectral features. Second, rather than relying on external SNR ground truth, the network self-estimates an SNR-bin probability from the I/Q features and generates a channel-quality embedding that conditions the classifier, requiring no SNR label at inference. On the RadioML2016.10a and 10b benchmark datasets, MSTFFNet achieves overall accuracies of 67.33% and 70.87%, outperforming state-of-the-art methods by 3.53% and 5.33%, with improvements of 5.91% and 9.52% in the low-SNR regime. These results demonstrate improved recognition performance across the SNR conditions represented in the two synthetic RadioML2016 benchmarks, particularly at low SNR.

## 1. Introduction

RF signals in modern wireless systems are increasingly observed under non-cooperative conditions, where a receiver must identify the modulation scheme of received signals without prior coordination with the emitter. Low SNR, fading, and channel uncertainty severely degrade recognition reliability, making robust AMR essential for spectrum monitoring and cognitive radio applications [1].

AMR is a fundamental task in RF signal pattern recognition [2], aiming to identify the modulation scheme of a received signal under unknown or partially known channel conditions. Because the modulation type reflects the signal structure, spectral occupancy, and transmission mechanism, AMR provides valuable prior information for signal demodulation, spectrum monitoring, interference analysis, and electromagnetic environment awareness [3]. Received signals, however, are typically affected by noise, fading, frequency offset, and limited observation length; under low SNR, the discriminative modulation features may be severely attenuated, making reliable recognition difficult [4].

Conventional AMR methods can be broadly divided into likelihood-based (LB) and feature-based (FB) approaches. LB-AMR formulates modulation recognition as a hypothesis testing problem [5] and achieves reliable performance when the signal, noise, and channel models are accurately known, but it usually relies on prior knowledge and incurs high computational complexity [6]. FB-AMR relies on hand-crafted features such as higher-order cumulants [7], cyclostationary features [8], and spectral features, combined with conventional classifiers. The performance of such methods depends heavily on expert-designed features [9] and is sensitive to noise, fading, and parameter mismatch. Data-driven methods that automatically learn discriminative representations from received signals are therefore needed.

Deep learning-based AMR (DL-AMR) [10] has attracted increasing attention owing to its strong representation learning capability. Existing methods have explored a variety of network architectures, including convolutional neural networks (CNNs), recurrent neural networks (RNNs), convolutional-recurrent networks, attention mechanisms, and Transformer-related models. CNNs are adept at extracting local waveform patterns [11] but are limited in modeling long-range temporal dependencies [12]; RNNs capture sequential relations [13,14] but are generally more computationally expensive and weaker in local feature extraction. CNN-RNN hybrids combine local feature extraction with temporal modeling [15,16] but still offer limited characterization of global context. Attention and Transformer-based approaches enhance the modeling of global dependencies [17,18,19] but may introduce computational redundancy and still require local inductive biases to preserve fine-grained signal structure. No single existing architecture fully covers robust AMR in complex wireless channel environments, where low SNR, multipath fading, and resource constraints coexist.

Many studies have also enriched modulation features from the perspective of input representation. The raw I/Q signal preserves the original amplitude and phase but mainly describes temporal waveform variations and is sensitive to noise. Time-frequency representations [20], such as the STFT and wavelet features, provide spectral information of non-stationary RF signals, yet their performance depends on transform parameters and may lose fine-grained temporal detail. Other representations, such as constellation diagrams [21] and cyclic spectra [22], highlight specific signal properties but typically capture only partial aspects of the modulation. Multi-modality fusion methods combine I/Q signals with transform-domain features for complementary information [23,24]. Simple or late fusion, however, often fails to fully capture cross-domain correlations [25], especially under low SNR, where the effective information in each modality is jointly weakened.

Beyond network architecture and input representation, SNR information has also been incorporated into AMR at the optimization level. Zheng et al. [26] proposed DL-PR, a model-agnostic priori regularization method that exploits the SNR distribution of training samples to adjust the loss function and improve feature discrimination across different SNR conditions. However, DL-PR requires SNR information from dataset labels or a separate blind SNR estimator and uses it primarily as a training prior rather than as a sample-wise conditional representation for classification.

Although addressing a different task, recent CSI-free BER minimization for dynamic RIS-assisted broadcasting [27] highlights the practical value of learning-based communication methods without explicit channel knowledge, which also motivates MSTFFNet to derive an SNR-bin cue from the received I/Q features rather than relying on externally supplied channel-quality information at inference.

In practical wireless channels, signal quality may vary considerably because of mobility, multipath propagation, and fading, resulting in SNR-dependent changes in temporal, spectral, and time-frequency features [28]. To account for these variations, we use SNR-bin labels only as auxiliary supervision during training and introduce an SNR-bin self-estimation mechanism. During inference, the mechanism predicts a soft SNR-bin distribution directly from the I/Q features and converts it into a channel-quality conditional embedding. This embedding is concatenated with the fused I/Q-STFT representation, enabling the classifier to adapt its decision process to the estimated noise level without requiring external SNR information or directly suppressing the main signal features.

To address the above challenges, we propose an SNR-conditioned multi-scale time-frequency fusion network, named MSTFFNet, to improve AMR performance across varying SNR levels, particularly under low-SNR conditions. The model takes the raw I/Q samples and their STFT representation as dual-domain inputs. The I/Q sequence is processed by a multi-scale temporal encoder to extract amplitude-phase features from different receptive fields, while the STFT map is fed to a time-frequency encoder to capture spectral structure. The two encoded feature sequences are then aligned and fused at the token level, where each token represents a local temporal feature vector generated from the corresponding signal segment. The fused tokens are further enhanced through temporal context modeling and efficient attention interaction. Finally, the model self-estimates the SNR-bin probability from the I/Q features, generates a channel-quality conditional embedding, and concatenates it with the fused representation for classification.

The main contributions of this work are summarized as follows:

1. We propose MSTFFNet, an AMR framework that jointly exploits the raw I/Q signal, its STFT time-frequency representation, and a self-estimated SNR-bin conditional embedding to improve modulation recognition across varying SNR levels within the evaluated benchmark conditions.
2. We design a multi-scale time-frequency fusion architecture that captures complementary temporal and spectral features from dual-domain inputs, with a cross-modal contrastive objective that encourages alignment between the I/Q and STFT representations.
3. We propose a token-level fusion and context modeling strategy that integrates I/Q and STFT features before classification, enabling cross-domain interaction and global context aggregation with linear-complexity attention.
4. We construct an SNR-bin self-estimation conditional classification head that is supervised by SNR-bin labels during training yet requires no external SNR ground truth at inference, allowing the classifier to adaptively adjust its decision boundary according to channel quality.

These contributions form a unified processing chain: the dual-domain encoders extract complementary temporal and spectral features, the token-level fusion and context modeling modules integrate them into a discriminative representation, and the self-estimated SNR-bin embedding conditions the final classifier according to signal quality. Together, they support the overall goal of improving AMR performance across varying SNR levels, particularly at low SNR, without requiring external SNR information at inference.

## 2. Signal Model

In a typical wireless communication system, the receiver front end sequentially performs amplification, down-conversion, low-pass filtering, and analog-to-digital conversion, producing a discrete complex baseband sequence. Following the discrete-time complex baseband signal model commonly used in automatic modulation recognition [5,6], the received signal after propagation through the wireless channel is expressed as follows:

(1) $$\begin{array}{c} r\left(n\right)=A\left(n\right)e^{j\left(\omega n+\theta \right)}x\left(n\right)+v\left(n\right),\;\;\;n=0,1,\ldots ,N-1 \end{array}$$

Here, $n\in I_{N}=\{0,1,\ldots ,N-1\}$ is the discrete-time sample index, and $N\in N^{+}$ denotes the observation length, i.e., the number of complex baseband samples contained in one signal example, rather than the number of signal examples. In this study, N=128. The symbol $j=\sqrt{-1}$ denotes the imaginary unit, $r\left(n\right)\in C$ and $x\left(n\right)\in C$ denote the received and transmitted complex baseband samples, respectively, and $A\left(n\right)\in R_{\geq 0}$ denotes the effective channel amplitude gain at the n-th sample. The parameters ω∈[−π,π) and θ∈[−π,π) denote the normalized angular frequency offset in radians per sample and the phase offset in radians, respectively.

The noise term $v\left(n\right)\in C$ is modeled as circularly symmetric complex additive white Gaussian noise independent of $x\left(n\right)$, i.e.,

(2) $$\begin{array}{c} v\left(n\right)∼CN\left(0,\sigma_{v}^{2}\right) \end{array}$$

The notation $\sigma_{v}^{2}$ denotes the complex noise variance,

(3) $$\begin{array}{c} \sigma_{v}^{2}=E\left[{\left|v\left(n\right)\right|}^{2}\right] \end{array}$$

where $\sigma_{v}$ is the corresponding standard deviation. Accordingly, the real and imaginary components of $v\left(n\right)$ each have a variance of $\sigma_{v}^{2}/2$.

The received complex sequence is converted into a two-channel real-valued I/Q representation by arranging its real and imaginary components as

(4) $$\begin{array}{c} X=\left[\begin{array}{ccc} Re\{r\left(0\right)\} & \cdots & Re\{r\left(N-1\right)\}\\ Im\{r\left(0\right)\} & \cdots & Im\{r\left(N-1\right)\} \end{array}\right]\in R^{2\times N} \end{array}$$

For N=128, the resulting I/Q input has a dimension of $X\in R^{2\times 128}$.

Although the I/Q sequence preserves the complete complex baseband waveform, it does not explicitly expose the local spectral evolution of the signal. A time-frequency representation can make modulation-dependent characteristics, such as the frequency transitions of FSK and the sideband structure of AM-SSB, more directly accessible to the network. Therefore, we apply the STFT to the received complex baseband sequence $r\left(n\right)$.

Specifically, the STFT uses a 64-point FFT, a 64-sample Hann window, and a hop size of H=2. The full complex STFT is defined as

(5) $$\begin{array}{c} Z\left(\tau ,f\right)=\sum_{m=0}^{N_{m}-1}r\left(m+\tau H\right)w\left(m\right)e^{-j2\pi fm/N_{m}} \end{array}$$

where $N_{m}=64$ is both the window length and the FFT size, H=2 is the hop size, $m\in \{0,1,\ldots ,N_{m}-1\}$ is the local sample index within each analysis window, τ∈{0,1,…,T−1} is the time-frame index, and $f\in \{0,1,\ldots ,N_{m}-1\}$ is the frequency-bin index of the full complex spectrum. The symmetric Hann window is defined as

(6) $$\begin{array}{c} w\left(m\right)=0.5-0.5\cos\left(\frac{2\pi m}{N_{m}-1}\right),\;m=0,1,\ldots ,N_{m}-1 \end{array}$$

During STFT computation, centering is disabled, and no samples are padded at either boundary. Only analysis windows that are completely contained within the original 128-sample observation are retained. Consequently, the number of time frames is

(7) $$\begin{array}{c} T=\left|\frac{N-N_{m}}{H}\right|+1=\left|\frac{128-64}{2}\right|+1=33 \end{array}$$

Because $r\left(n\right)$ is complex-valued, the one-sided STFT option is disabled, and the full $N_{m}=64$ frequency bins are first computed. Following the implemented preprocessing procedure, the frequency bins f∈{0,1,…,32} are then retained, giving F=33 frequency bins. The retained complex STFT representation is therefore $Z\in C^{F\times T}=C^{33\times 33}$, where the first dimension represents frequency and the second dimension represents time.

The retained complex STFT coefficients are represented in both Cartesian and polar forms. Specifically, their real part, imaginary part, magnitude, and phase are stacked along the channel dimension:

(8) S=StackRe{Z},Im{Z},Z,∠Z∈R4×33×33

Here, $\left|Z\right|\in R_{\geq 0}^{33\times 33}$ denotes the element-wise magnitude of the retained STFT coefficients, and $∠Z\in (-\pi ,\pi {]}^{33\times 33}$ denotes their wrapped principal phase. Phase unwrapping is not applied. The real and imaginary channels provide the Cartesian representation of the retained complex coefficients, whereas the magnitude and phase channels provide the corresponding polar representation. Together, the four channels provide complementary representations of the complex time-frequency spectrum for the subsequent encoder.

No logarithmic magnitude compression, sample-wise normalization, channel-wise normalization, resizing, or interpolation is applied before the four-channel STFT tensor is fed into the network. Feature scaling is subsequently performed by the batch-normalization layers in the STFT encoder.

Finally, MSTFFNet receives two complementary representations of each signal example: $X\in R^{2\times 128}$ for the original time-domain I/Q sequence and $S\in R^{4\times 33\times 33}$ for the corresponding time-frequency representation. The I/Q input preserves the original temporal waveform, whereas the STFT input explicitly describes its local spectral evolution.

## 3. Method

**Figure 1:**
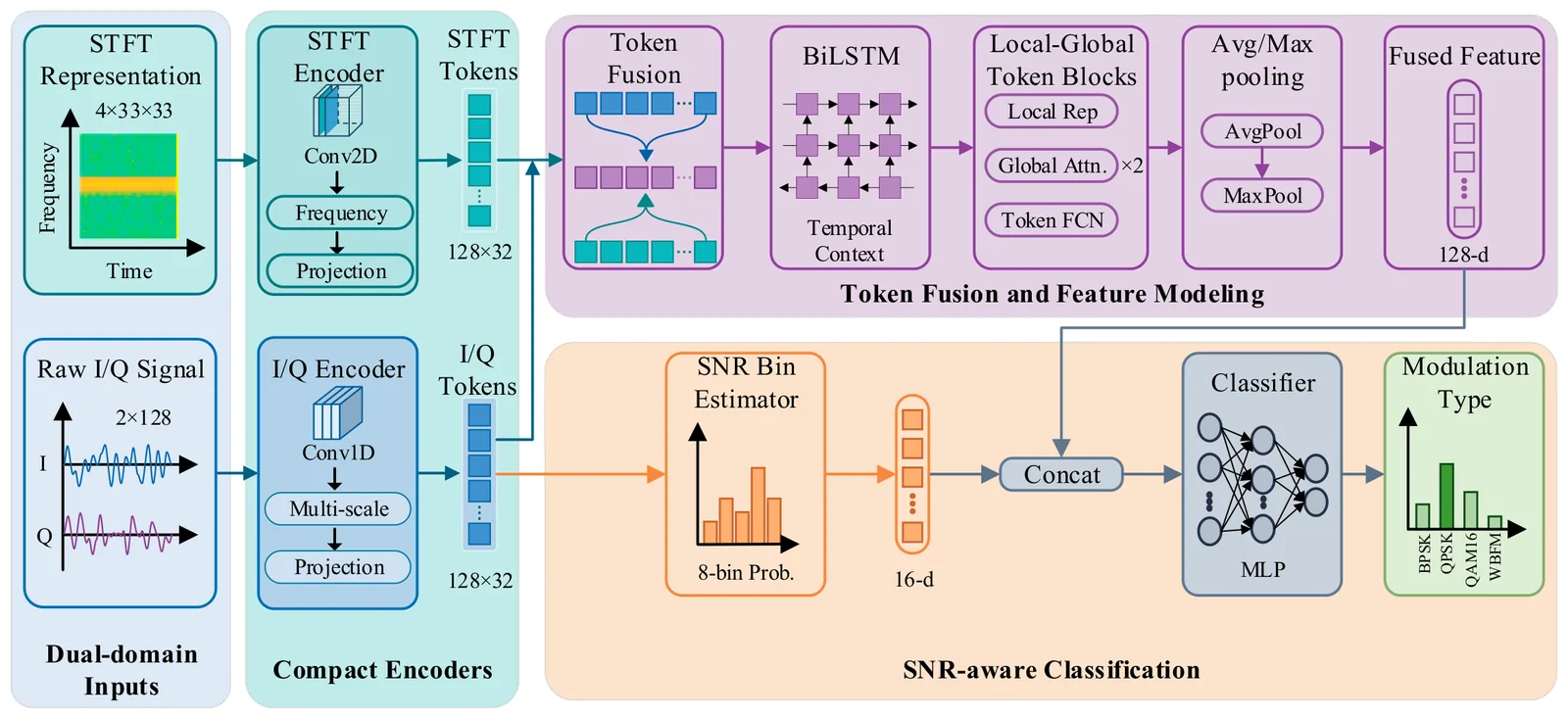
The overall network architecture of the proposed method.

AMR faces two structural difficulties. First, the features of different modulation types are distributed across different representation domains and time scales. For example, the instantaneous transitions of FSK, the constellation diagrams of QAM, and the envelope contours of analog modulation require different feature extraction methods. Second, the SNR in the dataset ranges from −20 dB to 18 dB, with a large variation in signal quality. At low SNR, the signal is almost drowned in noise, making it difficult for fixed-parameter classifiers to maintain optimal decision boundaries under all SNR conditions. To address these issues, this paper proposes a multi-scale time-frequency fusion network (MSTFFNet). The overall structure is shown in Figure 1, and the network consists of three modules: a dual-stream heterogeneous encoding module, a token-level fusion module, and an SNR interval self-estimation conditioned classification module.

### 3.1. Dual-Stream Heterogeneous Encoding

#### 3.1.1. IQEncoder

**Figure 2:**
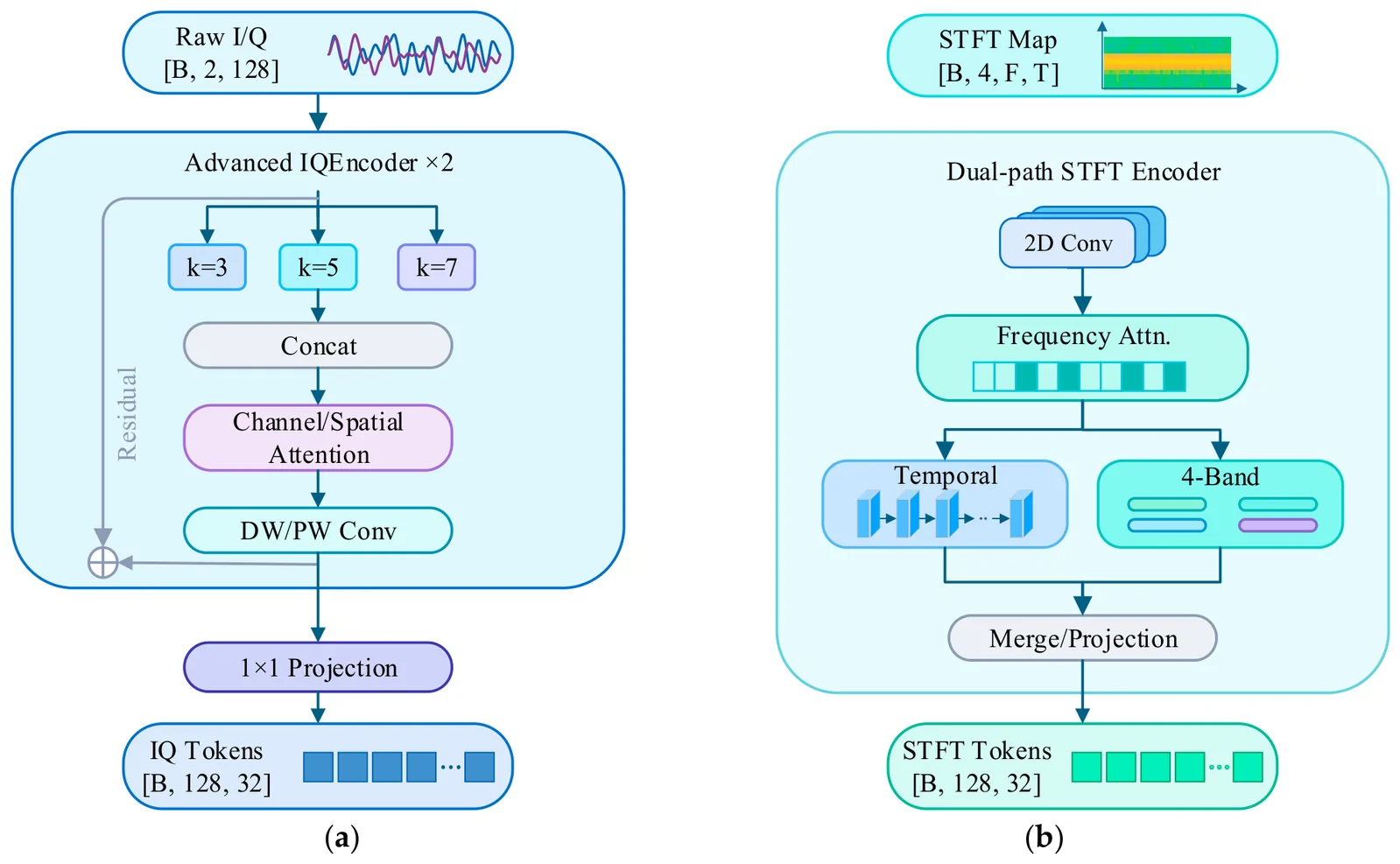
The structure of dual-stream encoding. (**a**) Structure of the IQEncoder. (**b**) Structure of the STFT encoder.

The modulation features in I/Q signals exist at multiple time scales. Standard convolutions with fixed kernel sizes are limited by a single receptive field and cannot effectively capture such multi-scale features. Therefore, this paper designs Advanced IQEncoder, which realizes multi-scale feature extraction through multi-branch parallel convolution and dual attention. The structure is shown in Figure 2a.

Let the input feature be X. There are three parallel convolution branches with kernel sizes k∈{3,5,7}, each containing two one dimensional convolutions, followed by batch normalization and GELU [29] activation after each layer. The outputs of the three branches are concatenated along the channel dimension:

(9) $$F_{\mathrm{multi}}=Concat\left(F_{3},F_{5},F_{7}\right)\in R^{3C\times L}$$

Let $X\in R^{C\times L}$ denote the input feature sequence. Each multi-scale branch produces $F_{k}\in R^{C\times L}$, where k denotes the corresponding convolutional kernel size. The branch outputs are concatenated along the channel dimension.

Then, channel attention and spatial attention [30] are applied sequentially. For channel attention, average pooling and max pooling are performed on the global time information simultaneously, and after passing through a shared two-layer fully connected network, they are added to generate channel weights. For spatial attention, the mean and max values are taken along the channel dimension and then concatenated. A one-dimensional convolution with a kernel size of 7 maps them to time weights. These scaled features are added to the identity mapping:

(10) $$F_{\mathrm{out}}=DWConv\left(A_{c}⊙\left(A_{s}⊙F_{\mathrm{multi}\;}\right)\right)+{Conv1d}_{1\times 1}\left(X\right)$$

where $A_{c}\in R^{3\times 1}$ and $A_{s}\in R^{1\times L}$ denote the channel- and spatial-attention weights, respectively, and ⊙ represents element-wise multiplication. During multiplication, $A_{c}$ is broadcast along the token dimension, whereas $A_{s}$ is broadcast along the channel dimension. DWConv$\left(\cdot \right)$ consists of a depthwise group convolution followed by a 1×1 pointwise convolution, which compresses the concatenated multi-scale representation from 3C to 2C channels. The residual projection maps the input to the same 2C×L dimension and facilitates direct gradient propagation across the multi-scale branches. Consequently, the output feature satisfies $F_{\mathrm{out}}\in R^{2C\times L}$.

The entire IQEncoder is composed of two Advanced IQEncoder stages in series. The base channel numbers are 64 and 128, respectively, and between stages, the time dimension is compressed by 2× max pooling. Finally, a 1×1 convolution projects the 256-dimensional features into an I/Q token sequence $T^{\mathrm{IQ}}\in R^{D\times L}$, where D=128 and L=32.

#### 3.1.2. STFT Dual-Path Encoder

The structure is shown in Figure 2b. As described in Section 2, the input I/Q signal is preprocessed by an STFT to yield a four-channel time-frequency map $S\in R^{4\times 33\times 33}$. The front end of the STFT encoder comprises two successive two-dimensional convolutional layers that progressively downsample the representation, increasing the channel dimension from 4 to 64 and then to 128, while two 2×2 max pooling operations compress the spatial dimensions to 9×9. This is followed by a two-dimensional residual block and a frequency attention gate, which averages the temporal dimension to produce a frequency descriptor and then applies a 1×1 convolution followed by a sigmoid to generate per-frequency channel-selective weights.

When the two-dimensional feature map is compressed into a one-dimensional token sequence, simply averaging along the frequency axis is robust for most modulation types, but it discards the structural contrast between frequency bands—a shortcoming that is particularly detrimental for WBFM and AM-SSB, whose spectra are continuously distributed. We therefore adopt a dual-path parallel strategy.

Let $F\in R^{C_{s}\times F_{c}\times T_{c}}$ denote the output feature map of the STFT convolutional encoder, where $C_{s}$, $F_{c}$, and $T_{c}$ represent the number of channels, frequency positions, and temporal positions, respectively. In the present implementation, $C_{s}=128$. Let P=4 denote the number of adaptively pooled frequency bands, D=128 the token dimension, and L=32 the output token length. The batch dimension is omitted from Equations (11)–(13) for clarity. For an STFT input of size 4×33×33, the convolutional encoder produces $F_{c}=T_{c}=9$.

Path A performs global average pooling along the frequency dimension. The resulting $C_{s}\times T_{c}$ feature sequence is linearly interpolated from $T_{c}$ to L temporal positions and projected from $C_{s}$ to D channels.

(11) $$T_{temp}={Proj}_{A}\left[{Interp}_{T_{c}\to L}\left(\frac{1}{F_{c}}\sum_{f=1}^{F_{c}}F_{:,f,:}\right)\right]\in R^{D\times L}$$

Path B applies adaptive average pooling to obtain P frequency bands while retaining the temporal dimension. The pooled-frequency and channel dimensions are then merged, interpolated to L token positions, and projected from $C_{s}P$ to D channels.

(12) $${\tilde{F}}_{\mathrm{band}}=\mathrm{Reshape}\left[\mathrm{AA}P_{P\times T_{c}}\left(F\right)\right]\in R^{\left(C_{s}P\right)\times T_{c}}\;T_{\mathrm{band}}={\mathrm{Proj}}_{B}\left[\mathrm{Inter}p_{T_{c}\to L}\left({\tilde{F}}_{\mathrm{band}}\right)\right]\in R^{D\times L}$$

The two token sequences are concatenated along the channel dimension, resulting in a 2D×L representation. A 1×1 convolution subsequently reduces the channel dimension from 2D to D.

(13) $$T_{STFT}={Conv}_{2D\to D}^{1\times 1}\left[{Concat}_{c}\left(T_{temp},T_{band}\right)\right]\in R^{D\times L}$$

Here, ${Interp}_{a\to b}\left(\cdot \right)$ denotes one-dimensional linear interpolation from sequence length a to b; ${AAP}_{P\times T_{c}}\left(\cdot \right)$ denotes adaptive average pooling to $P\times T_{c}$; $Reshape\left(\cdot \right)$ merges the channel and pooled-frequency dimensions; and ${Concat}_{c}\left(\cdot \right)$ denotes concatenation along the channel dimension. ${Proj}_{A}$ and ${Proj}_{B}$ are independent 1×1 convolutional projections.

The dual-path design allows the network to learn, during training, the degree to which each modulation type relies on the two paths: PSK-type signals favor the temporal-mean path, whereas analog modulations exploit the structural information retained by the frequency-band path.

### 3.2. Token Fusion and Refinement

Both encoders produce token sequences in $R^{D\times L}$, where D=128 and L=32. Rather than flattening the features into a single vector at the end of the encoders before fusion—the conventional paradigm—we fuse at the token level, thereby preserving the locality of the L temporal positions:

(14) $$T^{fused}=Conv{ld}_{2D\to D}\left(Concat\left(T^{IQ},T^{STFT}\right)\right)\in R^{D\times L}$$

A 1×1 convolution performs an adaptive linear combination of the two modal features at each temporal position, implicitly learning a position-wise confidence allocation between the two modalities. In contrast to multiplicative gating which drives the gating value towards zero at low SNR and thereby suppresses the entire feature pathway, linear fusion is stable to train and introduces no additional dependence on an SNR prior. After token fusion, a single bidirectional LSTM layer [31] captures temporal dependencies across positions. The phase reversals of PSK are instantaneous and local, whereas the frequency deviations of FM extend continuously over multiple symbol periods; the gating mechanism of the LSTM is naturally suited to such diverse temporal patterns. The hidden dimension is set to 64, and after bidirectional concatenation the output dimension is maintained at D=128. The fused tokens are then transformed by two local–global interaction modules, each comprising three serial submodules: local representation, efficient additive attention, and a token-wise feed-forward network. The local representation uses depth-wise separable convolutions [32] to capture local patterns in the token neighborhood, paired with a learnable LayerScale factor [33] to stabilize optimization in deeper layers. The additive attention replaces the $O\left(N^{2}\right)$ complexity of standard self-attention with $O\left(N\right)$ linear complexity; its core operations are as follows:

(15) $$G=\sum_{i=1}^{L}Softmax{\left(\frac{Q_{i}^{⊤}w_{g}}{\sqrt{D}}\right)}_{i}\cdot Q_{i}$$

(16) $$AddAttn\left(X\right)=W_{o}\left(G⊙K+Q\right)$$

where Q and K are the query and key obtained by linear projection of the input followed by $l_{2}$ normalization, $w_{g}\in R^{D}$ is a learnable parameter, and $W_{o}$ is the output projection matrix. The global context vector G is shared across the entire sequence, endowing each token position with a global receptive field. The token-wise feed-forward network is implemented by a 1×1 convolution with an expansion ratio of 4.

The refined token sequence is compressed along the temporal dimension via parallel adaptive average pooling and max pooling; the two outputs are concatenated and projected linearly to yield a 128-dimensional global feature vector $z\in R^{128}$.

### 3.3. SNR-Bin Self-Estimation and Conditional Classification

SNR is an important contextual prior in AMR. At high SNR, modulation-specific structures are clearly distinguishable, whereas at low SNR the within-class feature distribution spreads under noise and fading, requiring the classifier to adapt its decision boundary. Existing SNR-aware models often map an SNR value to multiplicative gates. When the estimated channel quality is poor, such gates may approach zero and suppress an already weak signal representation. Moreover, methods that require the true SNR at inference are difficult to deploy when channel-quality metadata are unavailable.

We adopt a different SNR strategy: rather than relying on an external SNR ground truth, we let the network estimate the SNR from the intermediate representation of the I/Q signal and inject the estimate into the classifier by concatenation. First, from the feature vector $z^{IQ}$ obtained by global pooling of the I/Q tokens, a lightweight classifier predicts the probability that the current signal falls into each SNR bin:

(17) $$p_{snr}=Softmax\left(W_{2}GELU\left(W_{1}z^{IQ}+b_{1}\right)+b_{2}\right)$$

where $z_{IQ}\in R^{128},\;W_{1}\in R^{64\times 128},\;b_{1}\in R^{64},\;W_{2}\in R^{M\times 64},\;b_{2}\in R^{M}$ and M=8. The interval set is {[−20,−15), [−15,−10), [−10,−5), [−5,0), [0,5), [5,10), [10,15), [15,20)} dB. The final interval contains the valid 16 dB and 18 dB samples in RadioML2016. A learnable embedding $e_{m}\in R^{16}$ is assigned to each bin, and the complete predicted distribution is converted into an SNR-conditional embedding by probability-weighted aggregation:

(18) $$h_{snr}=\sum_{m=1}^{M}p_{snr}^{\left(m\right)}\cdot e_{m}$$

This probability-weighted representation is denoted Predicted-soft throughout the experiments. It retains probability mass on adjacent intervals and permits a smooth transition near bin boundaries, avoiding the discontinuity introduced by an argmax assignment. At inference, $h_{snr}$ is computed entirely from the I/Q features via Equations (17) and (18), without recourse to any external SNR label.

The signal feature z and the estimated SNR embedding $h_{\mathrm{snr}}$ are concatenated along the feature dimension and fed into a two-layer MLP classifier:

(19) $$\hat{y}=W_{c2}GELU\left(Dropout\left(W_{c1}Concat\left(z,h_{snr}\right)+b_{c1}\right)\right)+b_{c2}$$

where $W_{c1}\in R^{128\times 144}$ and $W_{c2}\in R^{K\times 128}$. The fundamental distinction between concatenation-based injection and multiplicative gating is that every dimension of the signal feature z is preserved intact, with the SNR information acting solely as an additional conditional dimension. The classifier is thus free to learn which feature dimensions to emphasize under which SNR conditions, without the feature pathway being shut down entirely at low SNR. An auxiliary classifier $W_{aux}\in R^{K\times 128}$ is further introduced to predict directly from z; it participates only during training and serves as a deep supervision signal.

### 3.4. Training Objective

The total loss is a weighted sum of five terms:

(20) $$L=L_{cls}+\gamma L_{aux}+\alpha L_{con}+\beta L_{sup}+\eta L_{snr}$$

The classification loss $L_{\mathrm{cls}}$ is a 7:3 weighted mixture of cross-entropy and Focal Loss [34]. The cross-entropy term uses label smoothing [35] with a coefficient of ε=0.1 to suppress overfitting, while Focal Loss uses $\alpha_{f}=0.25$ and $\gamma_{f}=2$ to concentrate on hard to classify samples at low SNR. $L_{\mathrm{aux}}$ has the same form loss as the auxiliary classifier, with a weight of γ=0.3.

The cross-modal contrastive loss $L_{con}$ adopts InfoNCE [36] with a temperature coefficient of $\tau_{con}=0.07.$ The I/Q and STFT encoders produce normalized representations through independent projection heads; InfoNCE pulls together the two modal representations of the same sample while pushing apart those of different samples, thereby encouraging cross-modal alignment. The supervised contrastive loss $L_{sup}$ imposes intra-class compactness and inter class separation within each modality, with weights α=0.1 and β=0.05.

The SNR-bin estimation loss $L_{snr}$ takes the bin label $y_{bin}$, obtained by mapping the true SNR to its corresponding bin, as the supervision target and computes the cross-entropy against $p_{snr}$, with a weight of η=0.05. The key design here is that $y_{bin}$ is used only during training to supervise the SNR-bin classifier in Equation (17); at inference, $h_{snr}$ is produced entirely from the estimated probabilities $p_{snr}$, and the model accesses no SNR ground truth.

## 4. Experiment and Results

### 4.1. Dataset and Experimental Setup

We use the open source datasets RadioML2016.10a and RadioML2016.10b to evaluate the proposed AMR method. Both datasets are generated using GNU Radio and simulate modulated signals produced under complex channel conditions including AWGN Rayleigh fading, frequency offset, delay, and Doppler effects. RadioML2016.10a contains 11 modulation types: 8PSK, BPSK, QAM16, QAM64, QPSK, WBFM, CPFSK, GFSK, AM-DSB, AM-SSB, and PAM4—with SNR ranging from −20 dB to 18 dB in 2 dB steps, and 1000 samples per modulation type at each SNR, giving 220,000 samples in total. RadioML2016.10b is an extended version that increases the number of samples per modulation type at each SNR to 6000 and removes AM-SSB, yielding 1,200,000 samples in total. Both datasets use a signal format of 2×128, with each channel containing 128 sample points. For each modulation type, we randomly split the data into training, validation, and test sets in a 6:2:2 ratio within each SNR, ensuring a consistent distribution of SNR levels and classes across the subsets.

In all experiments, the STFT input uses a 64-point FFT, a 64-sample Hann window, a hop size of 2, no centering, no boundary padding, and a full complex STFT followed by the retention of frequency bins 0–32. No resizing, interpolation, additional normalization, logarithmic magnitude compression, or phase unwrapping was applied, resulting in a four-channel input tensor of size 4×33×33.

The model is implemented in Python 3.9 with PyTorch 1.12. The hardware comprises an Intel Core Ultra 9 285K CPU and an NVIDIA RTX 4090 GPU. Training runs for 100 epochs using the AdamW optimizer [37] with an initial learning rate of $10^{-3}$ and a weight decay of 0.01. The learning rate is scheduled by cosine annealing [38] with $T_{0}=20,T_{mult}=2$, and a minimum learning rate of $10^{-6}$.

Unless otherwise specified, all models in Table 1 were trained once using the same fixed training, validation, and test partitions. Because repeated training with multiple random seeds was not performed, the reported accuracy differences are interpreted as descriptive comparisons under the fixed experimental protocol rather than as evidence of statistical significance.

### 4.2. Comparison with Other SOTA Models

To evaluate the proposed method, we consider both modulation recognition performance and computational cost. Overall accuracy summarizes the aggregate recognition performance on the evaluated test sets, while SNR-specific accuracies characterize how the recognition performance varies across the SNR conditions represented in the two RadioML benchmarks, particularly in the low-SNR region. The computational metrics quantify the additional cost introduced by heterogeneous I/Q-STFT processing and characterize the empirical balance between recognition performance and inference cost under the specified hardware setting.

Let $N_{test}$ denote the total number of test samples, $y_{i}$ the true modulation label of the i-th sample, and ${\hat{y}}_{i}$ its predicted label. The overall accuracy is calculated over the complete test set as

(21) $${Acc}_{overall}=\frac{1}{N_{test}}\sum_{i=1}^{N_{test}}I\left({\hat{y}}_{i}=y_{i}\right)$$

where $I\left(\cdot \right)$ is the indicator function. For a specific SNR value s, the recognition accuracy is defined as

(22) $$Acc\left(s\right)=\frac{1}{N_{s}}\sum_{i\in D_{s}}I\left({\hat{y}}_{i}=y_{i}\right)$$

where $D_{s}$ is the subset containing the $N_{s}$ test samples at SNR s. The average accuracy over an SNR interval R is calculated as

(23) $$Acc_{R}=\frac{1}{\left|S_{R}\right|}\sum_{s\in S_{R}}Acc(s)$$

where $S_{R}$ denotes the set of evaluated SNR levels within interval R. In Table 1, the low-SNR and high-SNR intervals are defined as $\left[-20,\;0\right]$ dB and $\left[0,\;18\right]$ dB, respectively, with 0 dB retained as their common boundary reference. The highest accuracy is defined as the maximum recognition accuracy among all evaluated SNR levels:

(24) $${Acc}_{highest}=\underset{s\in S}{max}Acc\left(s\right)$$

Computational efficiency is evaluated using the number of trainable parameters, MACs, inference latency, throughput, and peak GPU memory. MACs represent the number of multiply-accumulate operations required for one forward pass. Inference latency is the average processing time per batch under the specified hardware and batch setting. Given a profiling batch size $B_{p}$ and an average batch latency $T_{\mathrm{batch}}$ in milliseconds, throughput is calculated as

(25) $$\mathrm{Throughput}=\frac{1000B_{p}}{T_{batch}}$$

Peak GPU memory denotes the maximum allocated device memory during inference. For MSTFFNet, the neural network inference cost and CPU-based STFT preprocessing cost are measured separately to distinguish model computation from input-generation overhead.

We compare the proposed method against six SOTA DL-AMR models, including GIGNet [39], AVGNet [40], FE-SKViT [41], MCLDNN [42], LSTM [13], and GRU [14], all of which are retrained and evaluated under the same data split and protocol.

Table 1 reports the results on the two datasets. With 1.98M parameters, MSTFFNet achieves the highest overall performance among the compared models. On RadioML2016.10a, its low SNR and overall accuracies exceed the best results of the compared methods by 5.91% and 3.53%, respectively. On RadioML2016.10b, the corresponding improvements reach 9.52% and 5.33%. MSTFFNet achieves a substantial performance advantage under the evaluated low-SNR conditions while maintaining performance comparable to the strongest baselines at high SNR. The small differences observed at high SNR are treated as descriptive results under the fixed single-run protocol.

To further evaluate computational efficiency, Table 1 reports the MACs, inference latency, throughput, and peak GPU memory. The executable models were profiled on an NVIDIA GeForce RTX 4090 with a batch size of 256, using 20 warm-up and 100 measured iterations. MACs were calculated using THOP. MSTFFNet requires 124.43 M MACs, fewer than GIGNet, AVGNet, and GRU. Its network inference latency is 24.79 ms per batch, with a throughput of 10,325 samples/s and peak GPU memory of 211.24 MB. CPU-based STFT preprocessing requires an additional 19.75 ms per batch, resulting in an approximate sequential end-to-end latency of 44.55 ms per batch and a throughput of 5747 samples/s. These results indicate that MSTFFNet incurs additional latency in exchange for improved low-SNR recognition. The linear-complexity property applies specifically to additive attention relative to quadratic self-attention, while the complete model achieves a practical balance between recognition performance and computational cost.

**Figure 3:**
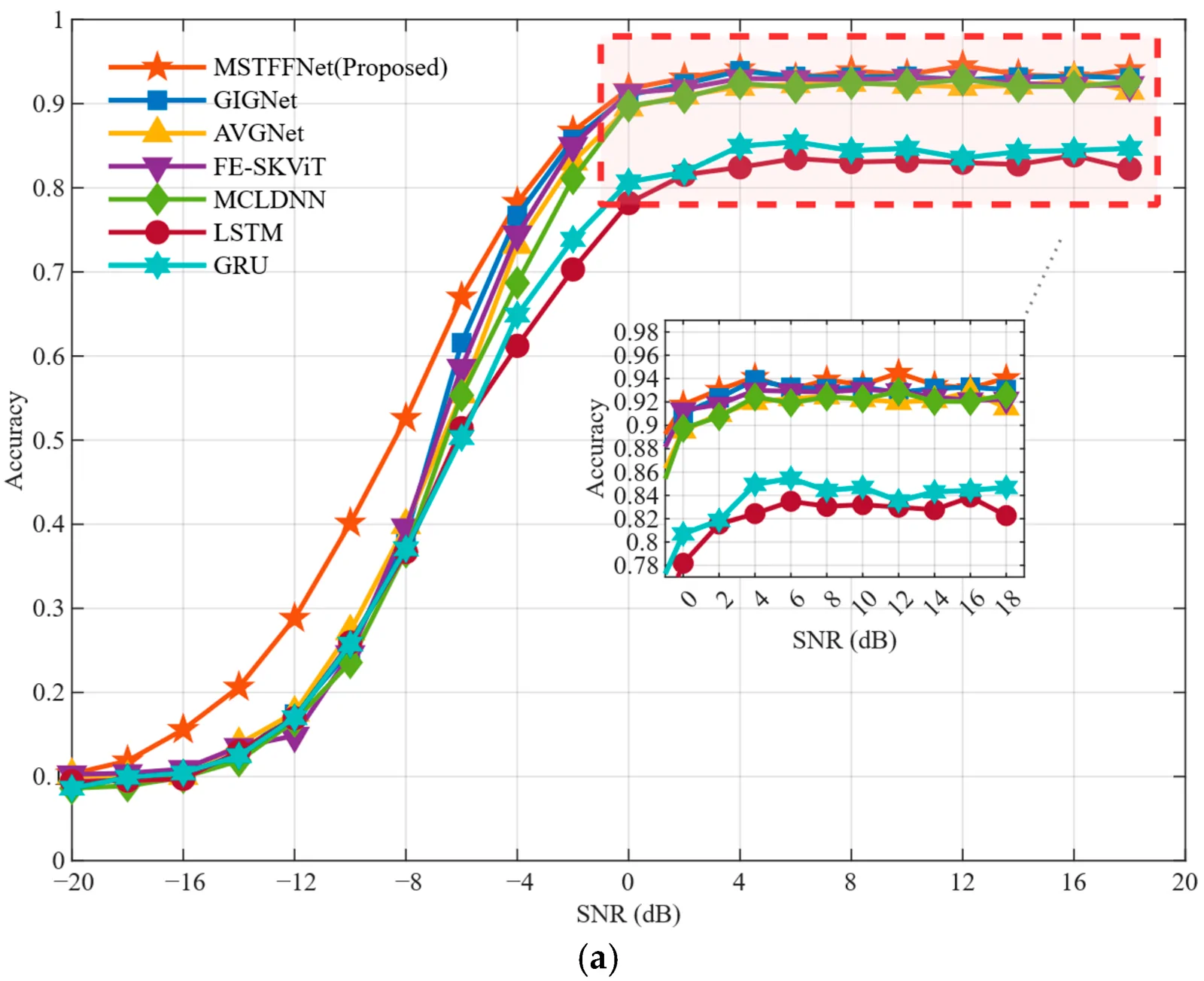

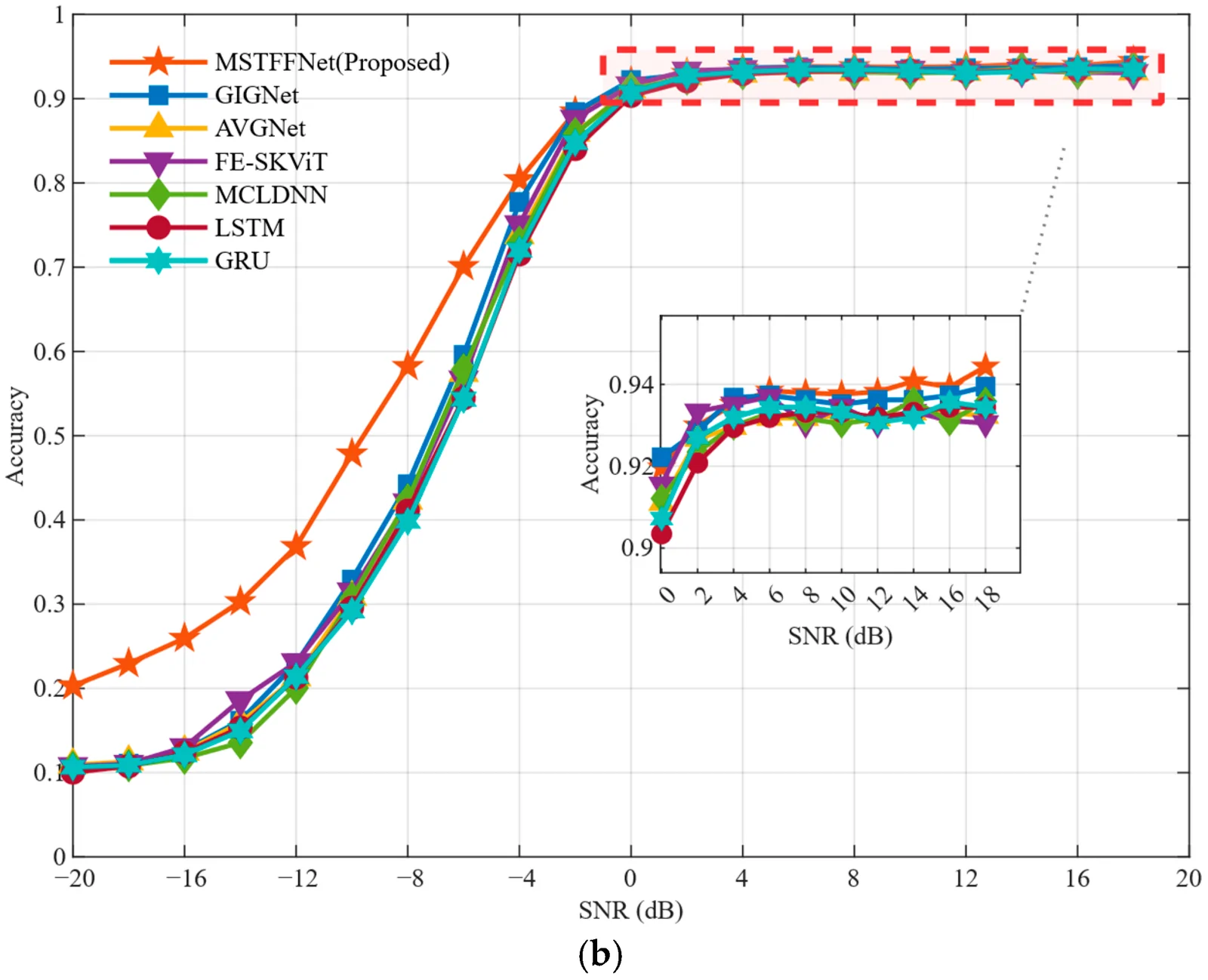
Recognition accuracy of MSTFFNet compared with other models on the RadioML2016.10a and RadioML2016.10b datasets. (**a**) RadioML2016.10a; (**b**) RadioML2016.10b.

Figure 3 shows the recognition accuracy of all models as a function of SNR on the RadioML2016 datasets in greater detail. At very low SNR (below −4 dB), MSTFFNet achieves a markedly higher recognition accuracy than the other models, which is the primary reason for its high overall accuracy. As the SNR increases, the features of different modulation types converge from being dispersed to being concentrated, making feature learning easier. At high SNR, the recognition curves gradually converge, and MSTFFNet maintains accuracy comparable to the strongest baseline models. This indicates that the SNR-conditioned classification approach has strong feature discriminating capability.

### 4.3. Modulation Analysis

**Figure 4:**
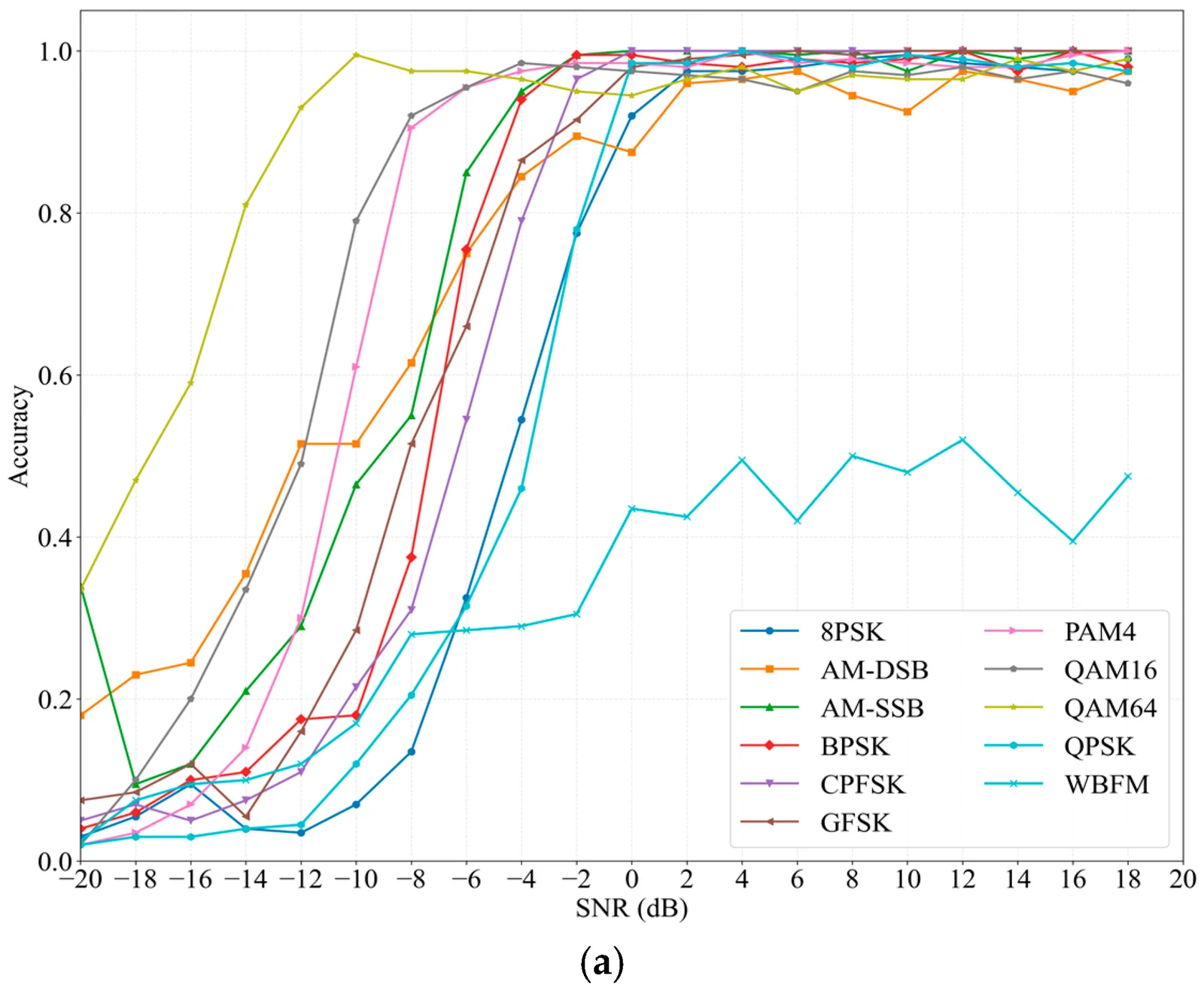

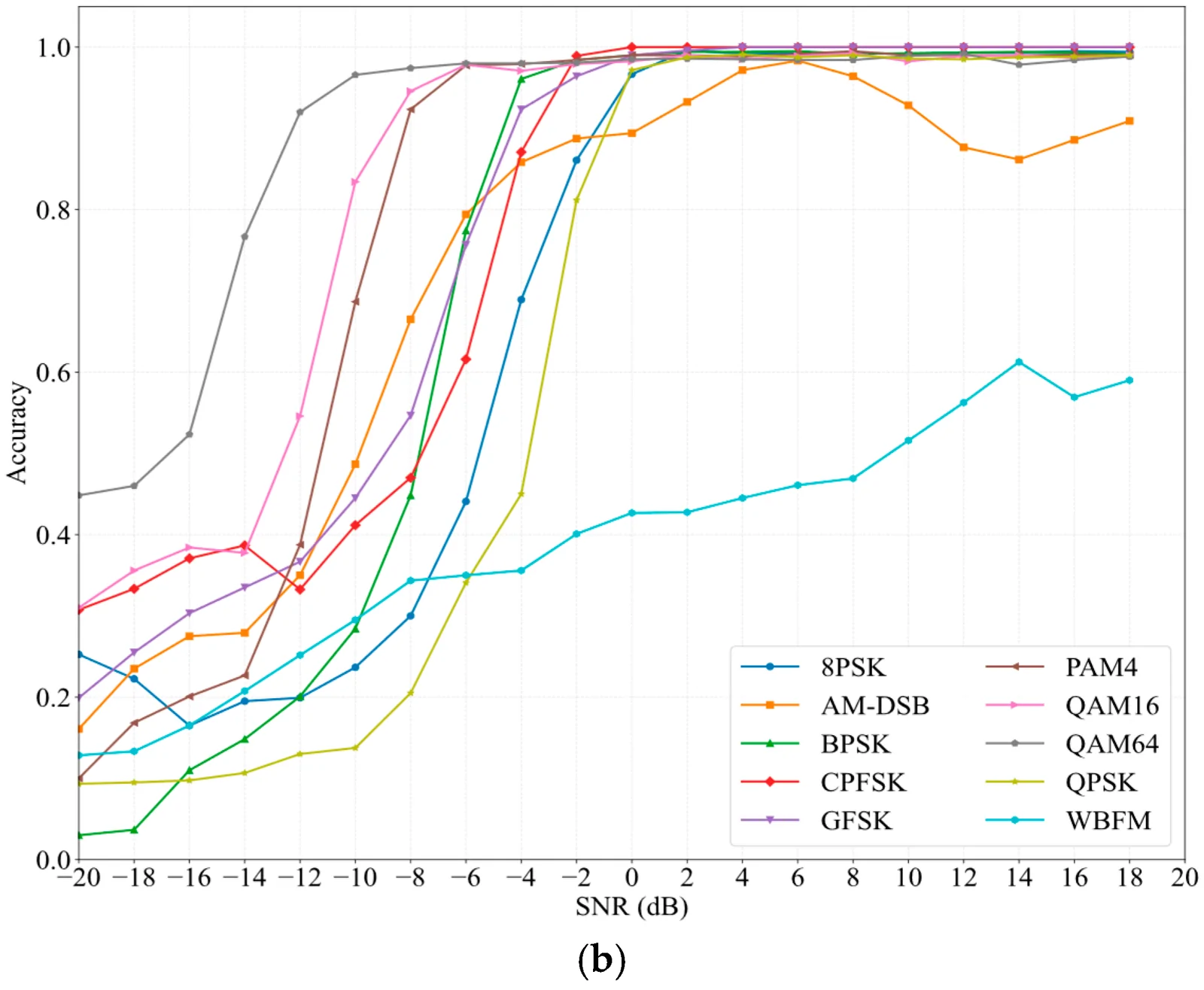
Recognition performance of different modulation types on the RadioML2016.10a and RadioML2016.10b datasets. (**a**) RadioML2016.10a; (**b**) RadioML2016.10b.

Figure 4 shows the recognition accuracy of MSTFFNet for each modulation type as a function of SNR on the two datasets. Overall, the recognition accuracy of all modulation types increases gradually with SNR. CPFSK and GFSK are almost perfectly recognized at high SNR, and PAM4 performs best in the medium-SNR range, indicating that the features of frequency modulations and simple amplitude modulations are relatively easy to learn. 8PSK, BPSK, and QPSK are nearly indistinguishable at low SNR, but their recognition rates rise rapidly as the SNR increases, reflecting the high sensitivity of phase information to noise.

WBFM deserves particular attention: its overall recognition accuracy is only 31.72%, and even in the high-SNR regime it remains below 50%, exhibiting a clear performance bottleneck. This phenomenon is not caused by noise alone; rather, the 128-point signal is too short to capture the continuous frequency variation of the wideband FM signal over time, causing its features to overlap heavily with those of AM-DSB.

**Figure 5:**
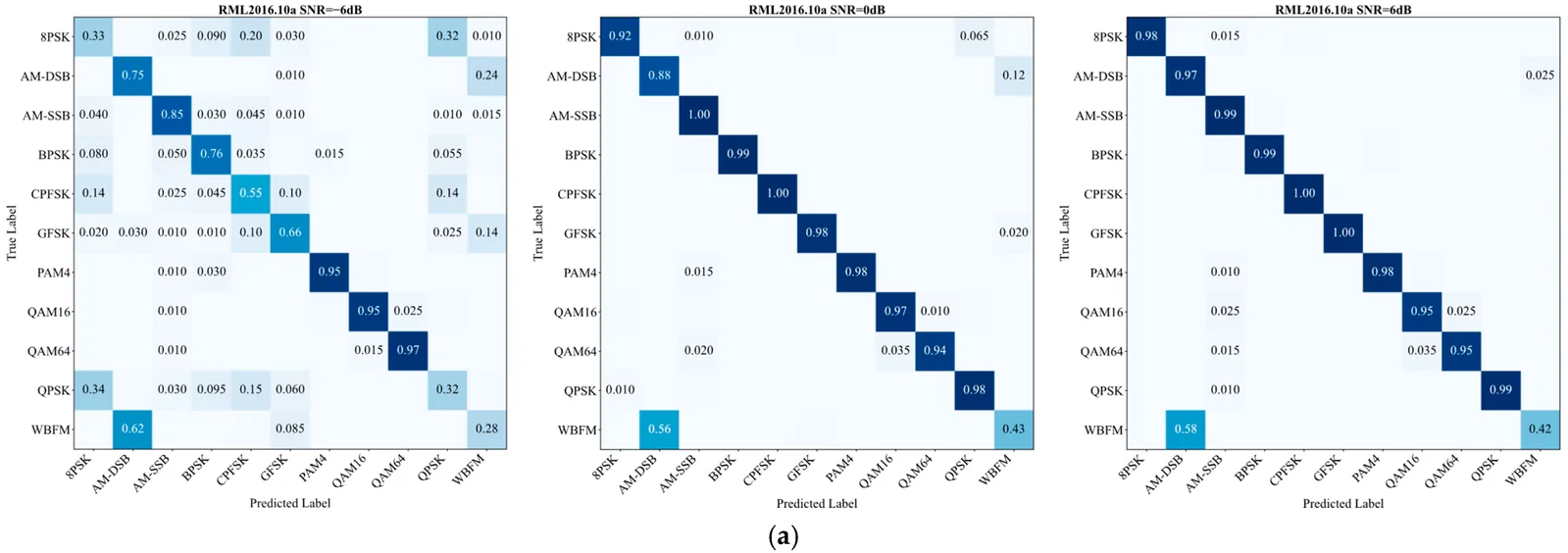

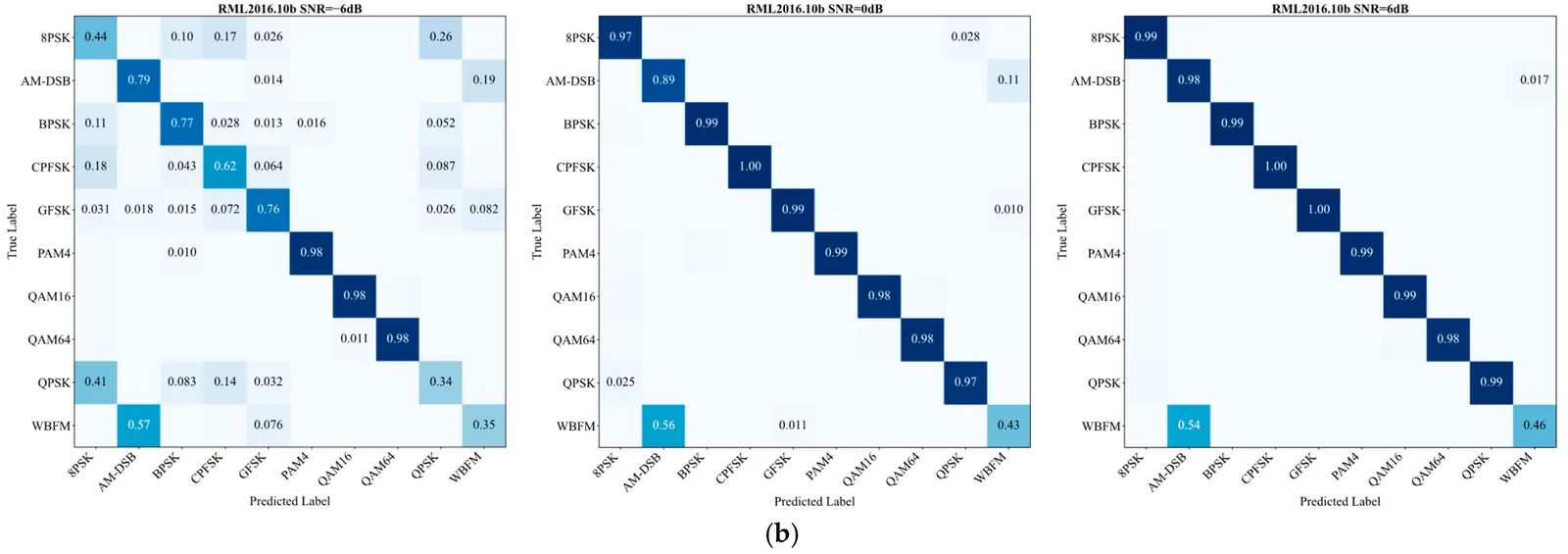
Normalized confusion matrices on the two datasets. (**a**) RadioML2016.10a (left: −6 dB, middle: 0 dB, right: 6 dB); (**b**) RadioML2016.10b (left: −6 dB, middle: 0 dB, right: 6 dB).

As shown in Figure 5, the confusion matrices reveal a structured pattern: all misclassifications occur within modulation families that are physically similar, rather than being randomly distributed across classes. The PSK family is severely confused at −6 dB but is largely separated once the SNR rises to 0 dB; the QAM family retains only minor residual confusion at 6 dB, and this does not spread to neighboring types such as PAM4 or GFSK. Had the model failed to learn an effective class structure, the misclassifications would be more randomly distributed rather than concentrated within physically related families. This result indicates that the dual-stream feature extraction of MSTFFNet has captured the hierarchical structure of the modulation types—by first discriminating the broad categories accurately and then refining the specific order within each family. PAM4 exceeds 95% on the diagonal at all SNR levels, and AM-SSB is almost never confused with any other class, further corroborating the model’s reliable capture of amplitude modulation and single-sideband spectral features.

### 4.4. Visualization Analysis

**Figure 6:**
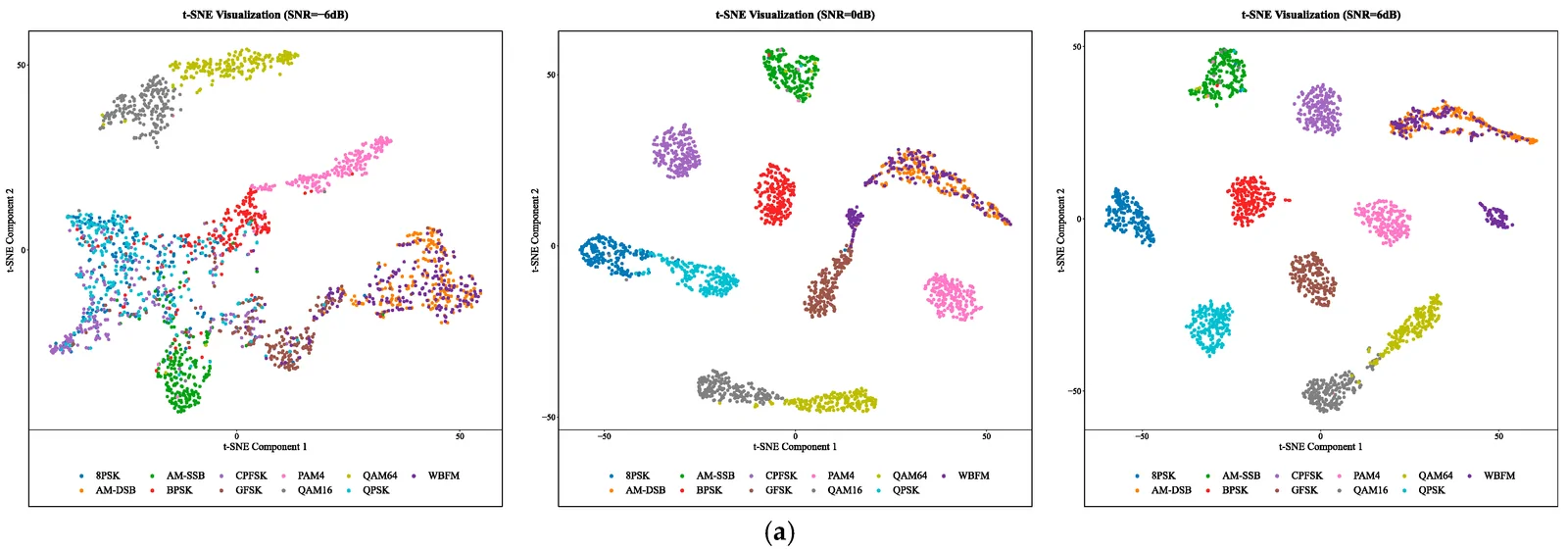

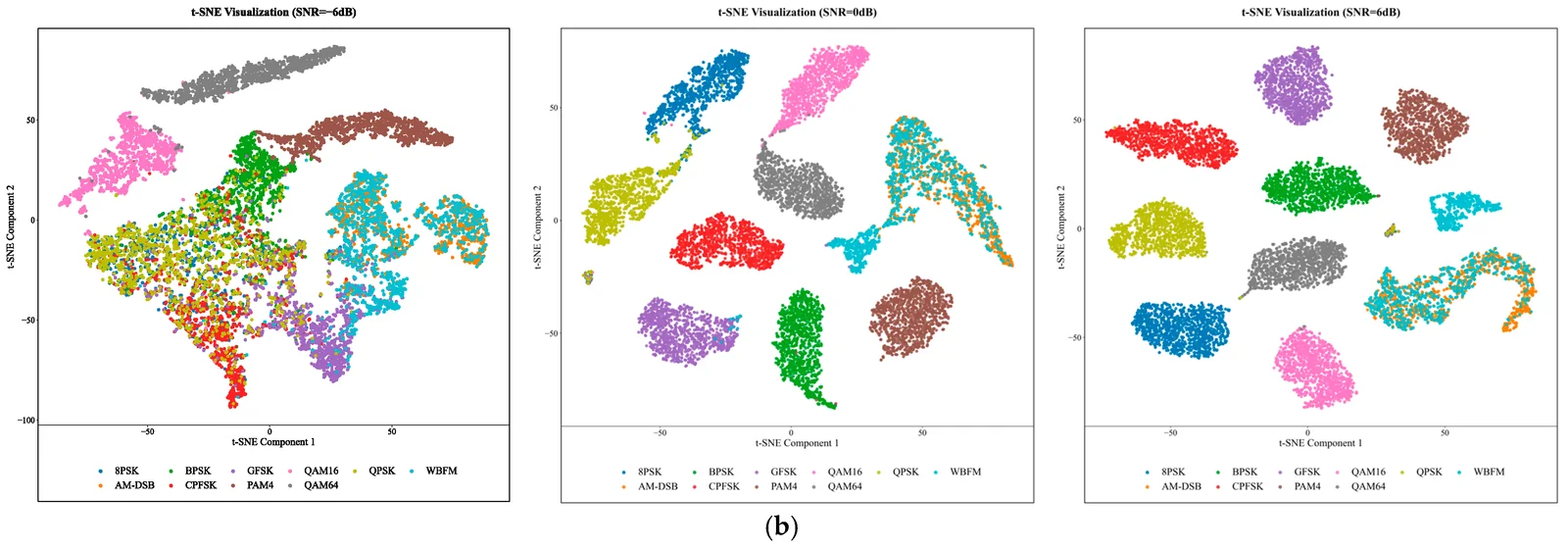
t-SNE visualization of the fully connected layer outputs. (**a**) RadioML2016.10a (left: −6 dB, middle: 0 dB, right: 6 dB); (**b**) RadioML2016.10b (left: −6 dB, middle: 0 dB, right: 6 dB). The horizontal and vertical axes represent t-SNE component 1 and t-SNE component 2, respectively, and their value intervals are indicated by the axis ticks. Both axes are dimensionless embedding coordinates without direct physical meaning and are used only to illustrate the relative neighborhood structure and class separation of the learned features.

Figure 6 shows the t-SNE [43] projections of the features learned by MSTFFNet at −6, 0, and 6 dB on the two datasets. At −6 dB, the scatter points of different classes are heavily intermingled, with almost no clear class boundaries; only PAM4 and AM-SSB show a faint tendency to cluster together. When the SNR rises to 0 dB, the situation improves markedly: 8PSK, BPSK, and QPSK each form independent clusters, QAM16 and QAM64, though still close to each other, begin to separate, and CPFSK and GFSK form compact blocks with well-defined boundaries. By 6 dB, with the exception of WBFM, all modulation types have formed independent clusters converging towards their own class centers, with clear boundaries between classes. This progressive convergence indicates that the model has learned the hierarchical structure of the modulation types—by first separating the broad categories and then refining the distinctions within each family.

WBFM and AM-DSB, however, are two exceptions. Even at the relatively high SNR of 6 dB, the scatter points of WBFM do not contract inwards as those of the other classes do; instead, they spread more widely and remain intertwined with the AM-DSB cluster, with no clear boundary ever emerging between the two. This trend is consistent across both datasets and shows no notable improvement even on the larger RadioML2016.10b dataset. This indicates that the problem lies not in insufficient data, but in an intrinsic bottleneck of the model’s representational capacity for wideband FM signals.

### 4.5. Ablation Studies

To verify the effectiveness of the key module designs in MSTFFNet, we conduct ablation studies along three dimensions: the input modality, the internal structure of the STFT encoder, and the SNR conditioning. All ablation studies keep the number of parameters consistent, ensuring that any performance difference is attributable solely to the structural choice.

#### 4.5.1. Input Modality Ablation

**Figure 7:**
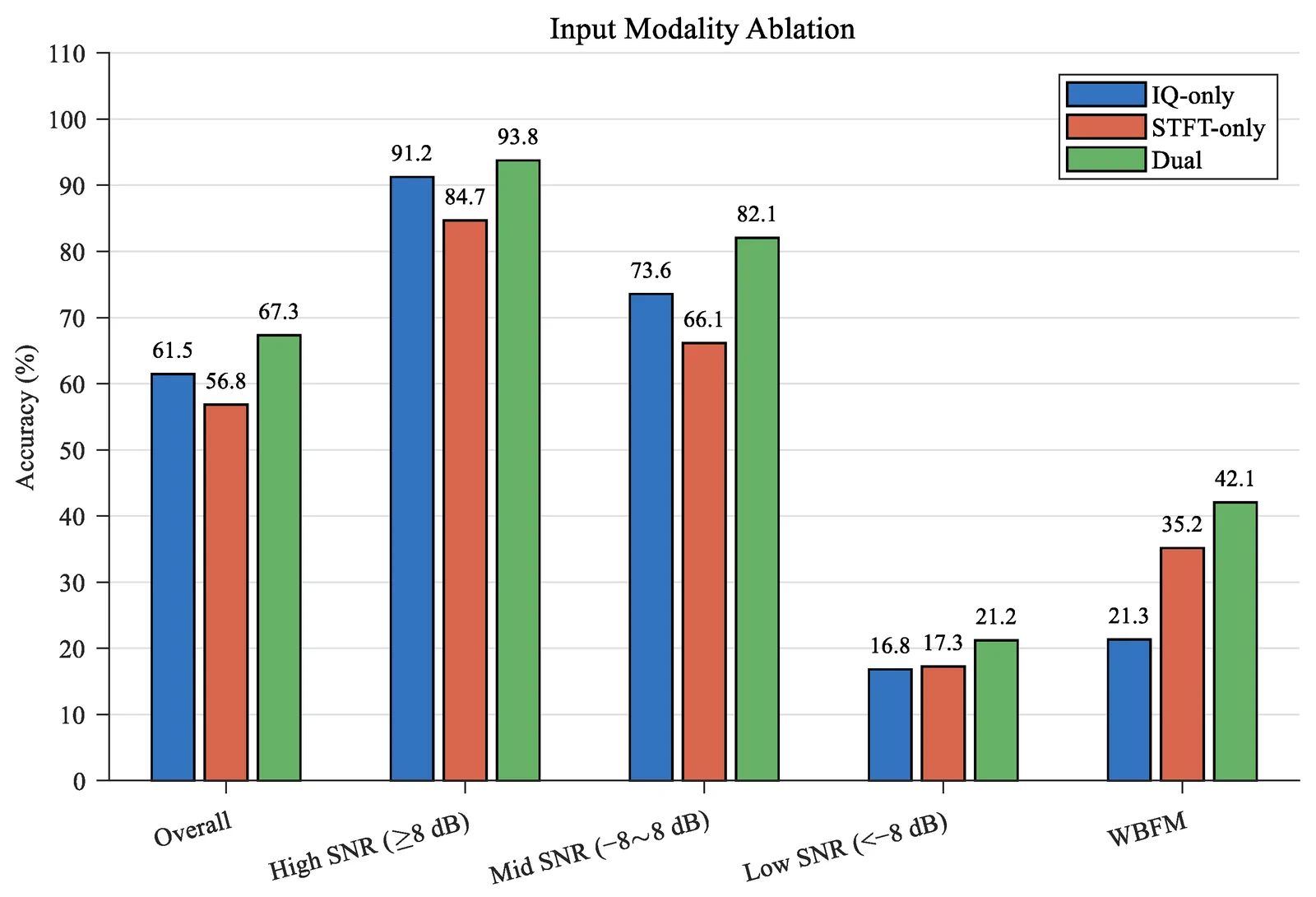
Results of the input modality ablation.

We first examine the actual contribution of the dual-modality fusion strategy to recognition performance. Three variants are designed: IQ-only, in which only the I/Q branch is retained and the STFT branch is replaced by a zero tensor; STFT-only; and the full dual-modality Dual. Their results on RadioML2016.10a are shown in Figure 7.

Three conclusions can be drawn from Figure 7. First, dual-modality fusion outperforms either single modality across all SNR ranges, with the overall accuracy improving over IQ-only and STFT-only by 5.85% and 10.51%, respectively, validating the effectiveness of complementary multi-modal information. Second, the two single modality branches exhibit a clear functional differentiation: IQ-only is close to Dual at high SNR, indicating that I/Q temporal features, when signal quality is sufficient, already support the recognition of most modulation types; STFT-only is weaker overall, but notably outperforms IQ-only on WBFM, showing that frequency-domain features play an irreplaceable role in distinguishing wideband FM signals—which also explains why dual-modality fusion can further raise the accuracy on this class to 42.08%. Third, the gap among the three variants narrows in the low-SNR range, where the information destruction by noise dominates across all modalities and the fusion gain is therefore limited.

#### 4.5.2. STFT Dual-Path Ablation

**Figure 8:**
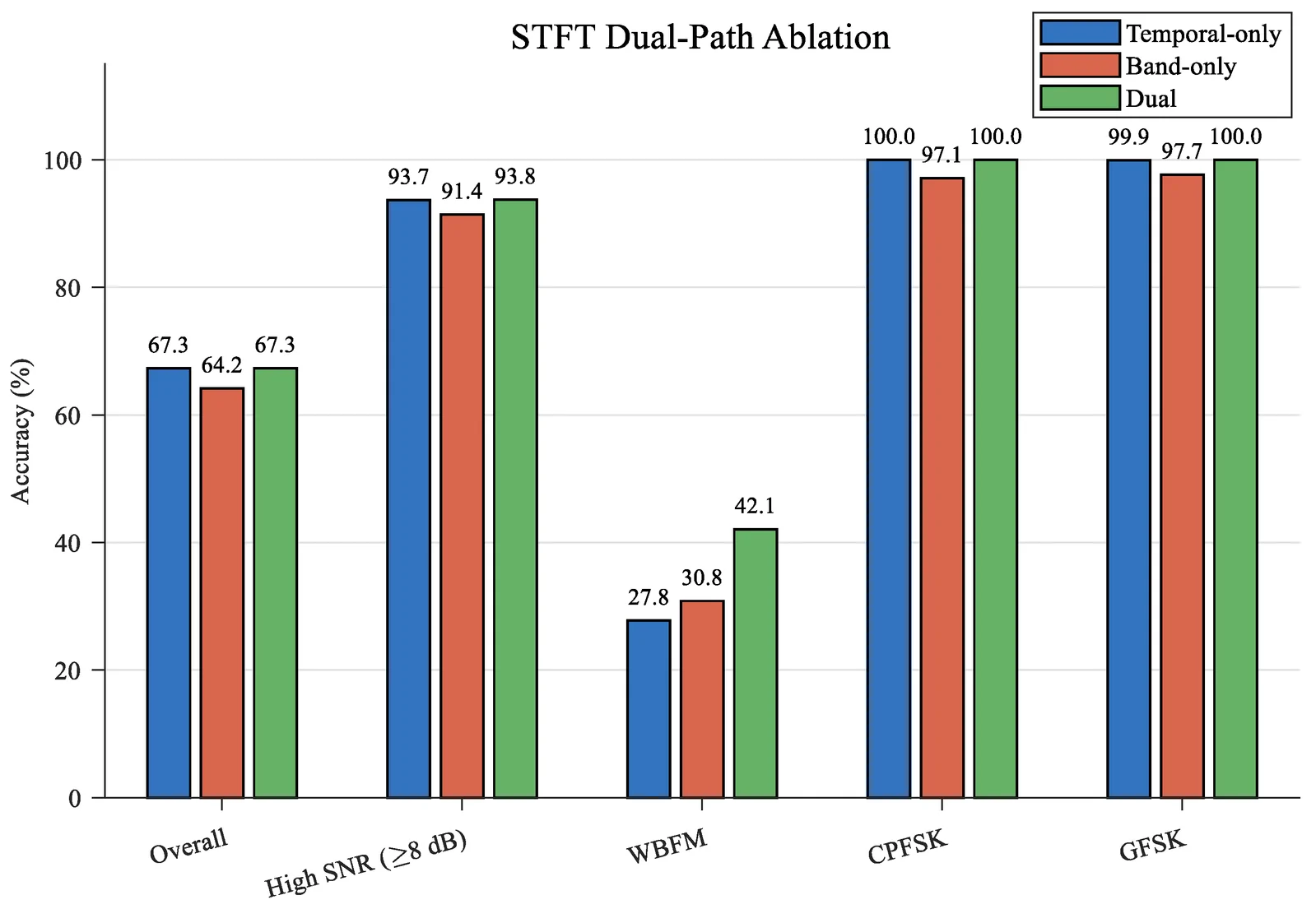
Results of the STFT dual-path ablation.

The STFT encoder employs two parallel paths internally: Path A (temporal-mean) averages along the frequency axis before projection, focusing on preserving temporal envelope variations; Path B (frequency band) retains the frequency-domain structure of 4 sub-bands through adaptive pooling, aiming to enhance the capture of frequency-domain details for wideband FM signals (WBFM). To examine the actual contribution of each path, we design three variants: temporal-only, band-only, and dual, and report their results on RadioML2016.10a in Figure 8.

In terms of overall accuracy, temporal-only is nearly on par with dual, whereas band-only drops by 3.18%. This indicates that, for most modulation types, the temporal-mean path already provides sufficient discriminative information, and the introduction of the frequency-band path may even slightly harm the recognition of other classes due to information dilution. On WBFM, however, the opposite is observed: temporal-only reaches only 27.75%, band-only improves slightly to 30.83%, and the dual-path combination surges to 42.08%, substantially surpassing either path used alone. This synergistic gain indicates that the two paths extract complementary frequency-domain representation dimensions—the temporal mean captures the envelope contour of WBFM, while the frequency-band structure preserves its sub-band energy distribution; only their combination constitutes a complete description of the wideband FM signal. This result also explains the core finding of the preceding visualization analysis: the recognition bottleneck of MSTFFNet on WBFM does not stem from a failure of the dual-path design, but rather from the insufficient representational capacity of the short STFT window for wideband FM signals.

#### 4.5.3. SNR Conditioning Ablation

MSTFFNet predicts the SNR-bin distribution from the I/Q features and injects the resulting condition into the classifier. To isolate the effect of the injection strategy, we compare three variants on RadioML2016.10a. Concat is the proposed Predicted-soft scheme: the eight-bin probability vector softly weights the learnable bin embeddings to form a 16-dimensional condition, which is concatenated with the fused feature. Gating uses the same self-estimation branch but maps the condition to channel-wise multiplicative gates. None removes the SNR branch and condition entirely.

Table 2 reports the results of the three strategies on RadioML2016.10a. Removing the SNR condition reduces overall accuracy by 2.98% and low-SNR accuracy by 4.38 points, whereas the high-SNR result changes only slightly. Multiplicative gating performs worst, particularly at low SNR, because it further attenuates the backbone representation. In contrast, Concat preserves the signal feature and supplies the estimated condition independently. The Concat row is the Predicted-soft mechanism defined in Section 3.3 and is the configuration used for the manuscript’s main results.

#### 4.5.4. Ablation of the Multi-Term Training Objective

A controlled ablation was performed on RadioML2016.10a with the same split, architecture, optimizer, schedule, batch size (256), training budget, and seed (42). The full objective used γ = 0.30, α = 0.10, β = 0.05, and η = 0.05 for $L_{aux}$, $L_{con}$, $L_{sup}$, and $L_{snr}$, respectively. Each term was removed individually by setting only its coefficient to zero. The I/Q and STFT projection heads were 128-128-128 with GELU and 128-dimensional normalized outputs, and τ = 0.07. Within each mini-batch, samples with the same modulation label were treated as positive pairs for supervised contrastive learning. For InfoNCE, the I/Q and STFT representations of the same sample formed a positive pair, while representations from different samples served as negatives. The smaller auxiliary weights keep these objectives subordinate to classification while providing representation regularization.

$$L=L_{cls}+\gamma L_{aux}+\alpha L_{con}+\beta L_{sup}+\eta L_{snr}$$

Removing $L_{snr}$ decreases the overall AMR accuracy from 67.33% to 66.87%, representing the largest reduction of 0.46%. This result confirms that SNR supervision contributes substantially to learning the channel-quality representation used by the conditioning branch. Removing $L_{con}$ causes the second-largest reduction of 0.28%, supporting its role in encouraging alignment between the I/Q and STFT representations. The removal of $L_{aux}$ and $L_{sup}$ reduces accuracy by 0.18% and 0.17%, respectively. Overall, the four loss terms provide complementary supervision and representation constraints. These single-run results are treated as controlled diagnostic evidence rather than tests of statistical significance.

The relatively small accuracy changes in Table 3 arise because the auxiliary objectives mainly provide training-time regularization. Removing an individual loss does not alter the deployed inference graph; therefore, the model parameters, MACs, latency, throughput, and peak inference memory remain essentially unchanged. Any computational saving is limited to the associated loss calculations and training-only heads. Removing $L_{\sup}$ causes the smallest accuracy decrease of 0.17%, while removing $L_{\mathrm{snr}}$ results in the largest decrease of 0.46%. Thus, omitting individual losses offers limited training-cost savings without reducing inference complexity. The complete objective is retained to achieve the best recognition accuracy.

### 4.6. Direct Evaluation of the SNR-Bin Self-Estimation Branch

To characterize both the reliability of the SNR self-estimation branch and the utility of its output for modulation classification, we evaluate the I/Q-derived SNR-bin probability vectors on the original held-out test splits. The analysis covers estimation accuracy and calibration, performance variations across true SNR levels and modulation classes, controlled interventions on the conditioning vector, and the propagation of SNR-bin estimation errors to AMR decisions.

#### 4.6.1. Direct Estimator Quality and Stratified Behavior

As shown in Table 4, the SNR-bin estimator is evaluated using seven complementary metrics. Top-1 accuracy denotes the proportion of samples for which the bin with the highest predicted probability exactly matches the true SNR bin. Macro-F1 is the unweighted average of the F1 scores calculated separately for all SNR bins, thereby reflecting class-balanced estimation performance. Within-one-bin accuracy measures the proportion of predictions whose argmax bin differs from the true bin by no more than one interval. MAE measures the mean absolute difference between the predicted argmax bin index and the true bin index. NLL evaluates the negative log-probability assigned to the true SNR bin, while the Brier score measures the squared difference between the predicted probability distribution and the one-hot target distribution. ECE-15 denotes the expected calibration error calculated over 15 confidence intervals and measures the weighted discrepancy between prediction confidence and empirical accuracy. Higher Top-1, Macro-F1, and within-one-bin values indicate better estimation performance, whereas lower MAE, NLL, Brier, and ECE-15 values indicate smaller estimation errors and better probability calibration.

**Figure 9:**
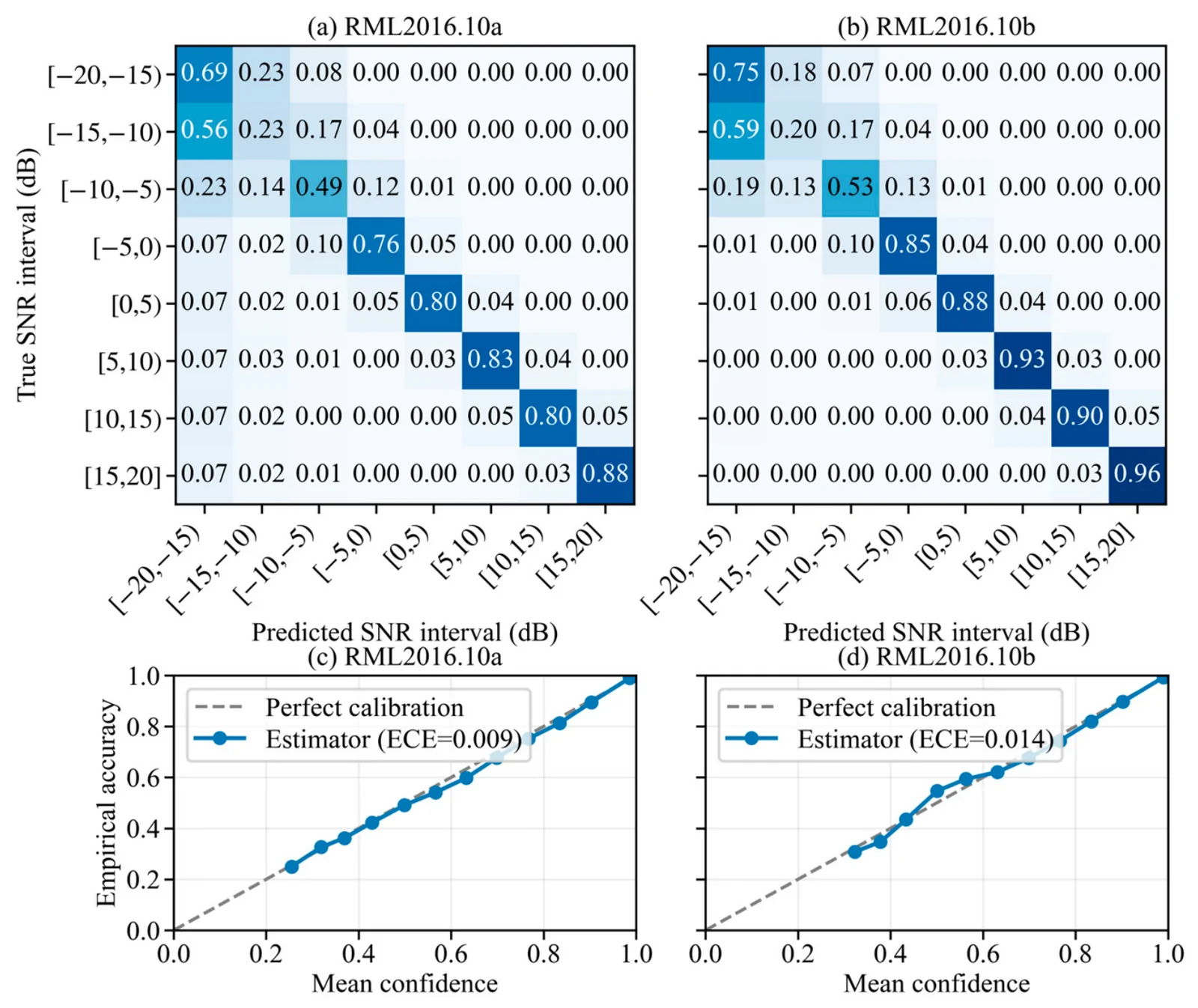
Direct evaluation of the SNR-bin estimator. (**a**) normalized SNR-bin confusion matrix on RML2016.10a; (**b**) normalized SNR-bin confusion matrix on RML2016.10b; (**c**) reliability diagram on RML2016.10a; (**d**) reliability diagram on RML2016.10b.

The branch achieves exact-bin accuracies of 68.60% and 75.47%, while the within-one-bin rates reach 88.80% and 94.87%. The low MAE values show that most errors are local, and the ECE-15 values indicate well-calibrated probabilities at the aggregate level. Figure 9 shows the corresponding row-normalized confusion matrices and reliability diagrams. The most difficult interval is [−15,−10) dB, with exact-bin recall of 22.93% and 20.20%; its mass is mainly distributed over neighboring bins rather than distant intervals.

Across true SNR levels, the exact-bin accuracy decreases around −14 to −10 dB, ranging from 22.36% to 29.23% on RML2016.10a and from 19.52% to 33.51% on RML2016.10b. It subsequently recovers from −8 dB upward and reaches 89.55% and 98.59% at 18 dB, respectively. The modulation-wise results show that AM-DSB and WBFM are among the easiest classes, achieving Top-1 accuracies of 81.83% and 82.60% on RML2016.10a and 83.32% and 83.21% on RML2016.10b, respectively. In contrast, QAM16 and QAM64 obtain lower exact-bin accuracies of 59.17% and 57.90% on RML2016.10a and 61.96% and 61.10% on RML2016.10b. AM-SSB is the main outlier on RML2016.10a, with a Top-1 accuracy of 13.98%. These results reveal modulation-dependent variations in SNR-bin estimation and identify the signal classes for which the estimated channel-quality distribution is less reliable.

#### 4.6.2. Matched Condition Interventions

As shown in Table 5, to separate valid sample-aligned channel-quality information from the effect of extra parameters, we intervene on the condition input while keeping the trained Predicted-soft checkpoint, fused feature, classifier, and 16-dimensional interface fixed. Oracle-bin replaces the predicted condition with the true one-hot bin; Predicted-hard uses only the argmax bin; Shuffled-soft permutes the predicted distributions across samples; Random-prior, Prior-mean, and Zero-hint provide matched-dimensional non-informative conditions.

Predicted-soft achieves overall accuracies of 67.33% and 70.87% on RML2016.10a and RML2016.10b, respectively, outperforming Predicted-hard by 10.084% and 6.415% and Shuffled-soft by 14.841% and 14.245%. These results demonstrate the importance of preserving the full probability distribution and sample-level alignment. Its advantages over Random-prior, Prior-mean, and Zero-hint further confirm that the improvement is not solely due to the additional conditioning dimensions.

To evaluate the potential benefit of ideal channel-quality information, we further construct an Oracle-bin diagnostic reference. Its AMR accuracy is slightly higher than that of Predicted-soft, indicating that accurate SNR-bin information can facilitate modulation recognition. Since the true SNR bin is generally unavailable at practical receivers, Oracle-bin serves only as an ideal diagnostic reference. In contrast, Predicted-soft is derived entirely from the I/Q samples and achieves performance close to Oracle-bin without requiring ground-truth SNR labels, demonstrating the practical value of the proposed method.

#### 4.6.3. Propagation of SNR-Bin Errors to AMR

As shown in Table 6, we group test samples by the absolute difference between the predicted argmax bin and the true bin, and we compare Predicted-soft with Predicted-hard within each group.

For RML2016.10a, soft conditioning improves AMR accuracy by 15.03% when the argmax error is one bin and by 30.67% when the error is two bins. The corresponding improvements on RML2016.10b are 19.53% and 31.12%, respectively. These results indicate that the uncertainty retained in the soft distribution remains informative even when the most probable SNR bin is incorrectly estimated. The ≥3 group on RML2016.10a has an atypical modulation/SNR composition associated with the AM-SSB failure mode; its raw accuracy should therefore not be interpreted as part of a monotonic trend. The within-group Soft-Hard gap provides a more reliable measure of robustness to SNR-bin estimation errors.

## 5. Conclusions

MSTFFNet is a multi-scale time-frequency fusion network for automatic modulation recognition under low-SNR conditions. It combines token-level fusion of raw I/Q signals and STFT representations with an I/Q-derived SNR-bin self-estimation branch, while an additive attention module captures global dependencies with linear computational complexity. On the RadioML2016.10a and 10b benchmarks, MSTFFNet achieves overall accuracies of 67.33% and 70.87%, respectively, outperforming the compared state-of-the-art methods. Ablation studies, controlled conditioning experiments, and error-stratified analyses verify the effectiveness of the dual-modality fusion, SNR-aware conditioning, and soft probability representation, while also demonstrating the robustness of the proposed method to SNR-bin estimation errors.

The present evaluation is limited to two closely related synthetic RadioML2016 benchmarks with in-distribution data splits and therefore does not establish cross-dataset generalization, robustness to unseen channel statistics, or deployment performance on resource-constrained hardware. Future work will focus on cross-dataset generalization, channel mismatch, hardware impairments, and validation with real over-the-air signal captures. Multi-scale time-frequency representations and self-supervised pre-training will also be explored to further improve generalization capability.

## Figures and Tables

**Table 1:** Performance comparison of different models on the two datasets (A: RadioML2016.10a, B: RadioML2016.10b).

Model	Dataset	Parameters	Highest Accuracy	SNR		Overall	MACs (M)	Latency (ms/Batch)	Throughput (Samples/s)	Peak Memory (MB)
				[−20, 0] dB	[0, 18] dB					
GIGNet	A	2.55M	93.90%	39.88%	92.92%	63.80%	286.40	36.82	6953	742.48
	B	2.71M	93.94%	42.59%	93.45%	65.54%	307.85	39.16	6537	786.23
AVGNet	A	1.83M	92.86%	39.10%	91.80%	62.93%	218.60	33.74	7587	698.91
	B	1.83M	93.31%	41.21%	92.93%	64.58%				
FE-SKViT	A	1.51M	93.06%	39.41%	92.45%	63.34%	34.25	4.280	59,813	184.57
	B	1.51M	93.70%	41.91%	93.11%	65.03%				
MCLDNN	A	0.41M	92.88%	37.37%	91.11%	62.02%	49.10	1.87	136,798	428.71
	B	0.41M	93.60%	40.70%	92.95%	64.30%				
LSTM	A	0.79M	83.85%	34.76%	82.38%	56.40%	101.98	4.91	52,182	281.41
	B	0.79M	93.44%	40.08%	92.86%	63.95%				
GRU	A	2.33M	85.46%	35.54%	83.90%	57.47%	304.09	5.94	43,121	486.47
	B	2.33M	93.56%	40.09%	93.01%	64.02%				
MSTFFNet (ours)	A	1.98M	94.50%	45.79%	93.45%	67.33%	124.43	24.79	10,325	211.24
	B	1.98M	94.44%	52.11%	93.61%	70.87%				

Note: “Highest Accuracy” denotes the highest single-point recognition accuracy across all SNR test points; “Overall” denotes the overall recognition accuracy. The intervals [−20, 0] dB and [0, 18] dB are independently reported operating ranges, and the result at 0 dB is included in both range-specific averages. All accuracy values are obtained from a single training run using the same fixed data partitions. Therefore, small differences between models, particularly those observed in the high-SNR region, should be interpreted descriptively and do not establish statistical significance.

**Table 2:** Ablation results of the SNR injection method.

Variant	Overall	High SNR [8, 18] dB	Mid SNR [−8, 8) dB	Low SNR [−20, −8) dB
None	64.35%	92.78%	78.65%	16.84%
Gating	63.19%	91.91%	77.53%	15.35%
Concat	67.33%	93.75%	82.07%	21.22%

Note: The low-, medium-, and high-SNR intervals are defined as [−20, −8) dB, [−8, 8) dB, and [8, 18] dB, respectively. Therefore, −8 dB is included in the medium-SNR interval, whereas 8 dB is included in the high-SNR interval.

**Table 3:** Loss-function ablation on RadioML2016.10a.

Variant	γ	α	β	η	Accuracy (%)
Full objective	0.30	0.10	0.05	0.05	67.33
w/o $L_{aux}$	0	0.10	0.05	0.05	67.15
w/o $L_{con}$	0.30	0	0.05	0.05	67.05
w/o $L_{sup}$	0.30	0.10	0	0.05	67.16
w/o $L_{snr}$	0.30	0.10	0.05	0	66.87

**Table 4:** Overall SNR-bin self-estimation performance.

Dataset	Test Samples	Top-1 (%)	Macro-F1 (%)	Within-One-Bin (%)	MAE (bins)	NLL	Brier	ECE-15
RML2016.10a	44,000	68.60	69.05	88.80	0.581	0.783	0.390	0.009
RML2016.10b	240,000	75.47	74.25	94.87	0.306	0.533	0.310	0.014

**Table 5:** Controlled AMR accuracy and Predicted-soft gains.

Condition	10a Overall (%)	10b Overall (%)	10a Gain vs. Predicted-Soft (%)	10b Gain vs. Predicted-Soft (%)
Oracle-bin	67.87%	71.58%	+0.54%	+0.71%
Predicted-soft (proposed)	67.33%	70.87%	—	—
Predicted-hard	57.24%	64.45%	−10.08%	−6.42%
Shuffled-soft	52.48%	56.62%	−14.84%	−14.25%
Random-prior	48.80%	56.69%	−18.52%	−14.18%
Prior-mean	57.61%	64.51%	−9.71%	−6.36%
Zero-hint	47.01%	48.24%	−20.32%	−22.63%

**Table 6:** Propagation of SNR-bin error to AMR accuracy.

Dataset	Absolute Bin Error	Fraction (%)	Predicted-Soft AMR (%)	Predicted-Hard AMR (%)	Soft-Hard (pp)
RML2016.10a	0	68.60	73.05	71.72	+1.33
	1	20.20	47.13	32.10	+15.03
	2	5.74	44.52	13.85	+30.67
	≥3	5.46	94.09	13.95	+80.14
RML2016.10b	0	75.47	77.01	75.54	+1.47
	1	19.40	54.12	34.59	+19.53
	2	4.79	45.79	14.67	+31.12
	≥3	0.34	17.71	9.96	+7.75

## Data Availability

The RadioML2016.10a and RadioML2016.10b datasets are employed for experimental verification in this study. Both are publicly available benchmark datasets widely used for the development and evaluation of automatic modulation recognition methods. Further information regarding access to the datasets can be obtained from the authors by email at wws_hf0612@163.com.

## Abbreviations

The following abbreviations are used in this manuscript:

AMR	Automatic Modulation Recognition
AWGN	Additive White Gaussian Noise
BER	Bit Error Rate
BiLSTM	Bidirectional Long Short-Term Memory
CNN	Convolutional Neural Network
CR	Cognitive Radio
CSI	Channel State Information
DL-AMR	Deep Learning-Based Automatic Modulation Recognition
DL-PR	Deep Learning with Priori Regularization
ECE	Expected Calibration Error
FB-AMR	Feature-Based Automatic Modulation Recognition
FFT	Fast Fourier Transform
GELU	Gaussian Error Linear Unit
GRU	Gated Recurrent Unit
I/Q	In-Phase and Quadrature
InfoNCE	Information Noise-Contrastive Estimation
LB-AMR	Likelihood-Based Automatic Modulation Recognition
LSTM	Long Short-Term Memory
MACs	Multiply-Accumulate Operations
MAE	Mean Absolute Error
MLP	Multilayer Perceptron
NLL	Negative Log-Likelihood
RF	Radio Frequency
RIS	Reconfigurable Intelligent Surface
RNN	Recurrent Neural Network
SNR	Signal-to-Noise Ratio
SOTA	State of the Art
STFT	Short-Time Fourier Transform
THOP	PyTorch Operation-Counting and Profiling Library
t-SNE	t-Distributed Stochastic Neighbor Embedding
WBFM	Wideband Frequency Modulation

**Mathematical Notation**

Symbol	Definition
N	Number of samples in one time-domain I/Q observation
$N_{m}$	STFT window length and FFT size
H	STFT hop size
T	Number of STFT time frames
F	Number of retained STFT frequency bins
X	Time-domain I/Q input
Z	Complex-valued STFT coefficient matrix
S	Four-channel STFT representation
F	Feature map generated by the STFT encoder
$C_{s}$	Number of feature channels in the STFT encoder
$F_{c}$	Frequency dimension of the encoded STFT feature map
$T_{c}$	Temporal dimension of the encoded STFT feature map
P	Number of pooled frequency bands
D	Token feature dimension
L	Length of the token sequence
$T_{IQ}$	Token sequence generated by the I/Q encoder
$T_{temp}$	Token sequence generated by the temporal-mean path
$T_{band}$	Token sequence generated by the frequency-band path
$T_{STFT}$	Token sequence generated by the STFT encoder
$T_{fused}$	Fused I/Q and STFT token sequence
Q	Query feature matrix in the additive attention module
K	Key feature matrix in the additive attention module
$q_{i},k_{i}$	Query and key vectors at the i-th token position
$w_{g}$	Learnable global attention vector
g	Global feature vector in the additive attention module
$W_{o}$	Output projection matrix of the additive attention module
$p_{\mathrm{snr}}$	Predicted probability distribution over SNR bins
M	Number of SNR bins
$e_{m}$	Learnable embedding of the m-th SNR bin
$h_{\mathrm{snr}}$	Probability-weighted SNR condition
K	Number of modulation classes
$\hat{y}$	Predicted modulation logits
L	Total training objective
$L_{\mathrm{cls}}$	Main classification loss
$L_{\mathrm{aux}}$	Auxiliary classification loss
$L_{\mathrm{con}}$	Cross-modal contrastive loss
$L_{\sup}$	Supervised contrastive loss
$L_{\mathrm{snr}}$	SNR-bin estimation loss
γ,α,β,η	Weights of the auxiliary loss terms
Interp(·)	One-dimensional interpolation operator
AAP(·)	Adaptive average pooling operator
Reshape(·)	Feature reshaping operator
Concat(·)	Feature concatenation operator
Proj(·)	Feature projection operator
⊙	Element-wise multiplication
$N_{\mathrm{test}}$	Total number of test samples
$N_{s}$	Number of test samples at SNR s
$D_{s}$	Test subset corresponding to SNR s
$S_{R}$	Set of evaluated SNR levels in interval R
$I\left(\cdot \right)$	Indicator function
$B_{p}$	Batch size used for computational profiling
$T_{\mathrm{batch}}$	Average inference latency per batch
